# Comparing two simplified questionnaire‐based methods with 24‐h recalls for estimating fortifiable wheat flour and oil consumption in Mandaluyong City, Philippines

**DOI:** 10.1111/mcn.13486

**Published:** 2023-02-22

**Authors:** Valerie M. Friesen, Jody C. Miller, Ryan B. Bitantes, Maria F. D. Reario, Charles D. Arnold, Mduduzi N. N. Mbuya, Lynnette M. Neufeld, Frank T. Wieringa, Ame Stormer, Mario V. Capanzana, Carl V. D. Cabanilla, Georg Lietz, Marjorie J. Haskell, Reina Engle‐Stone

**Affiliations:** ^1^ Global Alliance for Improved Nutrition Geneva Switzerland; ^2^ Alimentation, Nutrition, Santé, UMR QualiSud French National Research Institute for Sustainable Development (IRD) Montpellier France; ^3^ UMR QualiSud, Université de Montpellier, Avignon Université, CIRAD, Institut Agro, IRD Université de La Réunion Montpellier France; ^4^ Department of Human Nutrition University of Otago Dunedin New Zealand; ^5^ Helen Keller International Malate Manila Philippines; ^6^ Department of Nutrition, Institute for Global Nutrition University of California, Davis California USA; ^7^ Global Alliance for Improved Nutrition Washington District of Columbia USA; ^8^ Department of Science and Technology Food and Nutrition Research Institute Taguig City Philippines; ^9^ Human Nutrition Research Centre, Population Health Sciences Institute Newcastle University Newcastle Upon Tyne UK; ^10^ Present address: Food and Agriculture Organization (FAO) Rome Italy

**Keywords:** children, dietary intake assessment, food consumption, food fortification, food frequency questionnaire, quantitative methods, women of childbearing age

## Abstract

Information on fortifiable food consumption is essential to design, monitor and evaluate fortification programmes, yet detailed methods like 24‐h recalls (24HRs) that provide such data are rarely conducted. Simplified questionnaire‐based methods exist but their validity compared with 24HRs has not been shown. We compared two simplified methods (i.e., a household food acquisition and purchase questionnaire [FAPQ] and a 7‐day semiquantitative food frequency questionnaire [SQ‐FFQ]) against 24HRs for estimating fortifiable food consumption. We assessed the consumption of fortifiable wheat flour and oil using a FAPQ and, for wheat flour only, a 7‐day SQ‐FFQ and compared the results against 24HRs. The participants included children 12−18 months (*n* = 123) and their mothers 18−49 years selected for a study assessing child vitamin A intake and status in Mandaluyong City, Philippines. For fortifiable wheat flour, the FAPQ estimated considerably lower mean intakes compared to 24HRs for children and mothers (2.2 vs. 14.1 g/day and 5.1 vs. 42.3 g/day, respectively), while the SQ‐FFQ estimated slightly higher mean intakes (15.7 vs. 14.1 g/day and 51.5 vs. 42.3 g/day, respectively). For fortifiable oil, the FAPQ estimated considerably higher mean intakes compared to 24HRs for children and mothers (4.6 vs. 1.8 g/day and 12.5 vs. 6.1 g/day, respectively). The SQ‐FFQ, but not the FAPQ, generated useful information on fortifiable food consumption that can inform fortification programme design and monitoring decisions in the absence of more detailed individual‐level data. Potential adaptations to improve the FAPQ, such as additional questions on foods prepared away from home and usage patterns, merit further research.

## Key messages

- Fortifiable (i.e., industrially processed) food consumption data are essential to design, monitor and evaluate fortification programmes, yet 24‐h recalls (24HRs) that provide such data are rarely conducted.
- Simplified methods, such as targeted food acquisition and purchase questionnaires (FAPQs) and semiquantitative food frequency questionnaires (SQ‐FFQs), exist but their validity compared to 24HRs has not been shown.
- Among Filipino children 12−18 months and their mothers, the SQ‐FFQ generated useful information on fortifiable wheat flour consumption while the FAPQ systematically underestimated wheat flour and overestimated oil.
- Further research could strengthen the FAPQ to better capture foods prepared away from home and usage patterns.

## Graphical abstract

Fortifiable food consumption data are essential to design, monitor and evaluate fortification programmes, yet 24‐h recalls (24HRs) that provide such data are rarely conducted. Simplified questionnaire‐based methods exist but their validity compared with 24HRs has not been shown. We compared two simplified methods (i.e., a household food acquisition and purchase questionnaire [FAPQ] and a 7‐day semiquantitative food frequency questionnaire [SQ‐FFQ]) against 24HRs for estimating fortifiable food consumption among Filipino children 12−18 months and their mothers and found that the SQ‐FFQ generated useful information on fortifiable wheat flour while the FAPQ systematically underestimated wheat flour and overestimated oil.

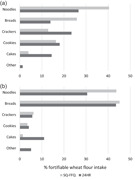

## 1 INTRODUCTION

Information on fortifiable (i.e., industrially processed) food consumption is essential to effectively design, monitor and evaluate large‐scale food fortification programmes (Allen et al., 2006). Different dietary assessment methods can be used to collect such data, but they vary in the level at which data are collected (i.e., household or individual), resource requirements and usefulness for informing fortification programme decisions (Coates et al., 2012). The 24‐h dietary recall method is commonly used to collect individual‐level data on total food and nutrient intakes (Wojtusiak et al., 2011). Such data are recommended to be collected during programme design to inform the selection of foods and fortification levels and during programme evaluation to assess impact on nutrient intakes (Coates et al., 2012). However, 24‐h recalls that collect such data are rarely conducted owing to concerns about technical and financial resources required (Fiedler et al., 2013). Alternative simplified methods for collecting fortifiable food consumption data that require less effort, time and cost to implement include targeted food acquisition and purchase questionnaires (FAPQs) and semiquantitative food frequency questionnaires (SQ‐FFQs). Targeted FAPQs collect household‐level data on acquisition and purchasing patterns for specific foods, which can be used to estimate individual‐level food intakes by applying the adult male equivalent (AME) method (Weisell & Dop, 2012). Targeted SQ‐FFQs collect individual‐level data on the frequency of consumption and portion sizes for specific foods over a defined time period (J. Cade et al., 2002). Examples of FAPQs include the relevant modules in Household Consumption and Expenditure Surveys (HCES) in low‐ and middle‐income countries (LMICs), which often collect information on commonly fortified staple foods (Fiedler et al., 2012). These data have been used to inform the selection of foods for fortification (Adams et al., 2022; Fiedler et al., 2008); however, they do not always distinguish between fortifiable and nonfortifiable forms of these foods (Fiedler, 2009). Additionally, targeted FAPQs for commonly fortified staple foods and a 7‐day SQ‐FFQ (for wheat flour only) are included in Fortification Assessment Coverage Toolkit (FACT) surveys (Friesen et al., 2019c). These data (along with data on micronutrient content of fortified foods) have been used to monitor fortification programme performance and potential for impact on nutrient intakes (Aaron et al., 2016, 2017; Friesen et al., 2020; Rohner et al., 2016).

The availability of fortifiable food consumption data for fortification programme decision making could be increased by incorporating simplified dietary assessment methods into existing surveys given their lower technical and financial resource requirements and/or enabling the use of secondary data from such simplified methods (e.g., from HCES or FACT surveys); however, evidence on the validity of simplified methods compared to 24‐h recalls is limited. Among studies that applied the AME method to HCES data to assess intake of potentially fortifiable staple foods compared with 24‐h recalls or other reference methods, wheat flour intake was consistently underestimated while agreement varied for other foods (Dary & Jariseta, 2012; Engle‐Stone & Brown, 2015; Lividini et al., 2013). Potential explanations for discrepancies between household FAPQ data (analysed with the AME method) compared to individual‐level intake data include inaccuracies in measurement of foods prepared away from home and household utilisation, limitations regarding the frequency of acquisition versus consumption, and inequitable intrahousehold distribution for the AME method. For SQ‐FFQs, the selection of appropriate food lists and methods of portion size estimation are likely to influence validity. To our knowledge, no studies have compared SQ‐FFQs against reference methods for assessing the intake of fortifiable foods specifically; however, among those that assessed the intake of general foods and/or food groups, agreement varied and context‐specific validation was recommended (J. E. Cade et al., 2004; Kolodziejczyk et al., 2012; Tabacchi et al., 2014).

We used data from a study that assessed vitamin A intake among Filipino children 12−18 months to estimate fortifiable food consumption among the children and their mothers (18−49 years) using three dietary assessment methods. These were: two simplified methods (i.e., a FAPQ and a 7‐day SQ‐FFQ) and a detailed dietary assessment approach, which included multiple 24‐h recalls and (among children only) observed weighed food records. In this article, we compare the results of the two simplified methods against those from the detailed dietary assessment as the reference method and discuss the utility of these methods to generate data for fortification programme decision making.

## 2 METHODS

### 2.1 Study design and participants

This study was part of a larger study that assessed usual dietary vitamin A intake among Filipino children receiving vitamin A supplementation (VAS) in Mandaluyong City in the National Capital Region of the Philippines. Children were categorised into one of three groups at the time of enrolment: (1) likely high retinol intake (>600 µg/day) and receipt of VAS in the past 30 days; (2) likely high retinol intake (>600 µg/day) and receipt of a VAS in the past 3−6 months; or (3) likely low/adequate retinol intake (200−500 µg retinol activity equivalents (RAE)/d) and receipt of VAS in the past 3−6 months. Sample size was based on the detection of mean retinol intake >600 µg/day in the high‐intake groups (i.e., Groups 1 and 2). Assuming 80% power, alpha 0.05, and 25% attrition rate, 50 children per group (*n* = 150 total) would be needed. A more detailed description of the study design and sample size is reported elsewhere (Engle‐Stone et al., 2022).

Mother−child pairs were prescreened at the time of the national VAS campaigns in March to May 2016 and September to November 2016. Study staff collected information on the child's receipt of VAS in the past 6 months from the master lists of children given VAS as part of the Government of the Philippines VAS programme and estimated vitamin A intake using a dietary screening questionnaire. Eligibility criteria included: (1) child 12−18 months of age, (2) mother 18−49 years of age, (3) mother and child living in the selected study communities for at least 1 year and planning to stay in the study area for the duration of the study, and (4) the child's receipt of VAS and estimated vitamin A intake were consistent with one of the groups described above. Exclusion criteria included: (1) child or mother had chronic disease or signs or symptoms of vitamin A deficiency, (2) the child had a weight‐for‐length *z* score < ‐2 compared to the WHO growth standards for the age and sex (de Onis et al., 2009), and/or (3) the mother was breastfeeding more than one child.

### 2.2 Data collection

In this study, we collected data on the consumption of fortifiable (i.e., industrially processed and not made at home) wheat flour and oil using three methods: a FAPQ, 7‐day SQ‐FFQ and a detailed dietary assessment approach, which included multiple 24‐h recalls and (among children only) observed weighed food records (Table 1). These foods were selected as there is mandatory legislation in place in the Philippines since 2000 that requires them to be fortified (Republic of the Philippines, 2000). Other data, including anthropometric measurements, blood samples and morbidity information from the child and breast milk samples from the mother, were collected for the main study and are described in detail elsewhere (Engle‐Stone et al., 2022).

**Table 1:** Characteristics of the food acquisition and purchase questionnaire (FAPQ), 7‐day semiquantitative food frequency questionnaire (SQ‐FFQ) and 24‐h recall (24HR).

Characteristic	FAPQ	SQ‐FFQ	24HR
Fortifiable food vehicle assessed^a^	Wheat flour, oil	Wheat flour	Wheat flour, oil
Level of data collection	Household	Individual	Individual
Recall period for data collection	Since the last time the food vehicle was purchased	Previous 7‐day	Previous 24‐h
Foods included	Fortifiable wheat flour and edible oil	25 commonly consumed food items that contain fortifiable wheat flour	All foods and beverages consumed
Specifies foods consumed (vs. food acquired or purchased for consumption)	No	Yes	Yes
Assesses mixed dishes or products containing the fortifiable food of interest	No	Yes	Yes
Accounts for foods consumed outside the home as well as at home	No	Yes	Yes

^a^ Fortifiable was defined as industrially processed and not made at home.

On Day 0 of the study, the mother of the child completed the FAPQ and SQ‐FFQ. The FAPQ collected household‐level information on general use and acquisition (e.g., gifts, food aid) and/or purchases of fortifiable wheat flour and oil (in their raw forms) along with information on the age and sex of all household members. The SQ‐FFQ collected individual‐level information on the consumption of fortifiable wheat flour‐containing foods in the last 7 days from a list of 25 items for both the child and the mother. For food items consumed, trained interviewers asked the mother to report the frequency of consumption over the past 7 days and estimate the usual portion size consumed using a photo album of various portion sizes for each food item. Fortifiable oil consumption was not assessed using the SQ‐FFQ method due to the difficulty of assessing amounts consumed in prepared foods obtained outside the household. The questionnaires were developed based on modules from the FACT household questionnaire template (i.e., the household roster and fortification coverage modules for the FAPQ and the individual consumption module for the SQ‐FFQ) (Friesen et al., 2019b). Questionnaires were adapted to the local context (i.e., response options and language) and, for the SQ‐FFQ, a list of commonly‐consumed, wheat flour‐containing foods and a photo album of portion sizes was prepared by the local field team according to the FACT guidelines (Friesen et al., 2019a).

Throughout the 28‐day study period, four 24‐h recalls were scheduled for the child and two for the mother on nonconsecutive days to capture both weekend and weekday intake. The 24‐h recall collected individual‐level data on total dietary intake. During each 24‐h recall, trained interviewers asked the mother to report all foods and beverages consumed in the past 24 h for either the child or herself. Interviewers used a multiple‐pass method and collected recipe information (or best estimation) for all items regardless of source (i.e., prepared at home vs. purchased) (Gibson & Ferguson, 2008). For any wheat flour and oil consumed or used to prepare foods, interviewers probed to capture additional details needed to determine whether the food item was fortifiable. In addition, one 12‐h observed weighed food record (with 12‐h recall of the previous night's consumption) was conducted for each child except in some cases where it was not possible due to security concerns or was inadvertently not conducted. In those cases, an additional 24‐h dietary recall was conducted instead, if feasible. In this analysis, all complete days of data for each child (whether from 24‐h recall or 12‐h observation plus 12‐h recall) were combined to estimate usual intake distributions. For simplicity, we refer to these data as 24‐h recalls.

### 2.3 Estimating fortifiable food consumption

The amounts of fortifiable foods consumed daily by the children and their mothers were estimated separately for each of the three dietary assessment methods, as follows.

#### 2.3.1 FAPQ

We first determined the daily amount of fortifiable food consumed by the household by dividing the reported amount the household obtained on the last occasion by the reported duration that this amount usually lasts in the household. We then applied the AME method put forth by Weisell and Dop (2012), which assumes that an individual's consumption of household food is proportional to their energy requirements (Food and Agriculture Organization FAO, 2003, 2004). First, we assigned each household member an age‐ and sex‐specific AME and then summed the AMEs together to produce a household AME. We then divided the individual's AME by the household AME and multiplied it by the daily amount of fortifiable food consumed by the household to estimate the amount of fortifiable food consumed by the target individual per day in grams. Individuals from households that reported not consuming a fortifiable form of the food vehicle were assigned zero for the amount of fortifiable food consumed. Because the AME method uses household‐level data to estimate individual‐level consumption, these estimates are typically referred to as ‘apparent consumption’ but are hereafter referred to as consumption for ease of comparison with the other methods.

#### 2.3.2 7‐day SQ‐FFQ

For the 25 food items in the SQ‐FFQ, the grams of food in each of the various portion size options in the photo album were measured during photo album development, and the grams of fortifiable wheat flour in each portion were determined based on food composition tables and nutrition labels for packaged foods. For each food item reported being consumed by the target individual, we multiplied the number of grams of fortifiable wheat flour in the portion size reported by the frequency the item was consumed per week and then divided by seven to estimate the amount consumed daily. We then summed all food items containing wheat flour for the individual per day to obtain a cumulative total of fortifiable wheat flour in grams per day. Any of the food items the individual reported not consuming were assigned zero for the grams consumed for the food item.

#### 2.3.3 24‐h recalls

All food and beverages reportedly consumed by the target individuals in the 24‐h recalls were converted into grams based on food composition tables and nutrition labels for packaged foods. The amounts of fortifiable wheat flour and oil in each food item or mixed dish (e.g., the amount of wheat flour in a given quantity of bread or biscuits) were calculated from recipe information. We then summed the resulting grams of fortifiable wheat flour and oil for all food items for each individual on each day to obtain the cumulative total grams of each fortifiable food consumed per person per day. We adjusted the values for within‐person variation to estimate usual intake distributions of fortifiable wheat flour and oil by applying the amount‐only National Cancer Institute method using the Simulating Intake of Micronutrients for Policy Learning and Engagement (SIMPLE) macro (Luo et al., 2021).

### 2.4 Data analyses

Data analyses were carried out in Stata version 15.1 (StataCorp LLC) and SAS version 9.4 (SAS Institute Inc.). We calculated means, medians, and percentiles of fortifiable food consumption estimates from the three dietary assessment methods. With the sample size of 123 participants, we were powered to be able to estimate mean consumption with a 95% confidence interval with ± 0.19 standard deviations, a relatively precise interval for interpreting average consumption. For the FAPQ and SQ‐FFQ methods, values >3 SDs from the mean were considered outliers and excluded (i.e., FAPQ: two children and three mothers for wheat flour, seven children and five mothers for oil; SQ‐FFQ: three children and one mother for wheat flour). Additionally, 24‐h recall observations that were missing a corresponding FAPQ or SQ‐FFQ observation for comparison were excluded (i.e., when matched to FAPQ: 9 children and 10 mothers for wheat flour, 14 children and 12 mothers for oil; when matched to SQ‐FFQ: 10 children and 8 mothers for wheat flour).

We qualitatively compared the distributions of fortifiable food consumed as estimated by the FAPQ and SQ‐FFQ methods with those from the 24‐h recall reference method. For wheat flour, we also examined and compared the food sources as a percentage of total fortifiable wheat flour consumption to understand potential sources of differences between the SQ‐FFQ and 24‐h recalls. We did not do formal statistical significance testing to compare the individual‐level estimates of consumption from the FAPQ and SQ‐FFQ methods with those from the 24‐h recalls because the 24‐h recall usual intake estimates are intended to be interpreted only at the population level. Moreover, the interpretation of similar data for the purpose of designing and monitoring food fortification programmes is typically done at the population level.

### 2.5 Ethics statement

This study was conducted according to the guidelines laid down in the Declaration of Helsinki and all procedures involving research study participants were approved by the Research Ethics Board of the University of the Philippines‐Manila, the Institutional Review Board of the University of California, Davis, and the Institutional Review Board of Newcastle University, and it is registered on Clinicaltrials.gov (NCT03030339). All study procedures were explained to mothers at the time of enrolment and the mother provided written informed consent for herself and the child to participate.

## 3 RESULTS

A total of 123 child−mother pairs were enroled in the main study. All 123 children and 117 mothers completed the 24‐h recalls, and 116 child−mother pairs completed the FAPQ and the SQ‐FFQ. On average, children were 14.4 months of age, mothers were 28 years of age, and 50% of children were breastfed at the time of the study. Additional child, maternal, and household characteristics are reported elsewhere (Engle‐Stone et al., 2022).

### 3.1 Fortifiable wheat flour and oil consumption

**Figure 1:**
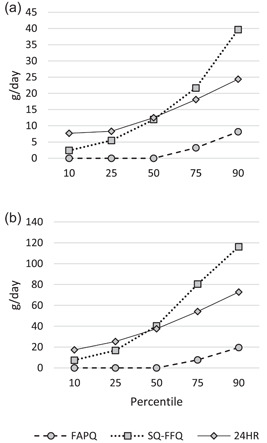
Distribution of the amount of fortifiable wheat flour consumed (g/day) among (a) children (12−18 months) (*n* = 114 for FAPQ; *n* = 113 for SQ‐FFQ and 24HR) and (b) their mothers (18−49 years) (*n* = 113 for FAPQ; *n* = 115 for SQ‐FFQ and 24HR) in Mandaluyong City, Philippines, estimated by FAPQ (household‐level assessment based on reported amount of fortifiable food the household obtained on the last occasion and duration that this amount usually lasts in the household and application of the adult male equivalent method), 7‐day SQ‐FFQ (individual‐level assessment based on reported portion size and frequency of consumption of a list of 25 common food items containing fortifiable wheat flour in the past 7 days), and 24HR methods (individual‐level assessment based on reported amount of fortifiable food consumed in the past 24 h adjusted for within‐person variation; mean 5.0 days of data per child (including one 12‐h observed weighed food record plus 12‐h recall in 57 children) and 2.0 days of data per mother). FAPQ, food acquisition and purchase questionnaire; SQ‐FFQ, semi‐quantitative food frequency questionnaire; 24HR, 24‐h recall.

**Figure 2:**
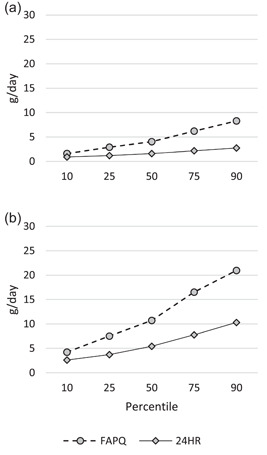
Distribution of the amount of fortifiable oil consumed (g/day) among (a) children (12−18 months) (*n* = 109) and (b) their mothers (18−49 years) (*n* = 111) in Mandaluyong City, Philippines, estimated by FAPQ (household‐level assessment based on reported amount of fortifiable food the household obtained on the last occasion and duration that this amount usually lasts in the household and application of the adult male equivalent method) and 24HR methods (individual‐level assessment based on the reported amount of fortifiable food consumed in the past 24 h adjusted for within‐person variation; mean 5.0 days of data per child (including one 12‐h observed weighed food record plus 12‐h recall in 57 children) and 2.0 days of data per mother). FAPQ, food acquisition and purchase questionnaire; 24HR, 24‐h recall.

The difference between the amounts consumed as estimated by the FAPQ and SQ‐FFQ methods compared to the 24‐h recalls varied depending on the method and fortifiable food (Table 2, Figures 1 and 2). For fortifiable wheat flour, the FAPQ mean and median intakes were more than six times lower than those from the 24‐h recalls for both children and mothers. Conversely, the SQ‐FFQ mean intakes were 11%−22% higher while median intakes were within 5%−7% compared to those from the 24‐h recalls. For the SQ‐FFQ method, there was greater variation in the distribution of fortifiable wheat flour intake among both children and mothers compared to the distribution estimated using 24‐h recalls. For fortifiable oil, the FAPQ mean and median intakes were 2−2.5 times greater than those from the 24‐h recalls and there was greater variation in the distribution of intake among both children and mothers.

**Table 2:** Amount of fortifiable wheat flour and oil consumed (g/day) by children and their mothers estimated by the FAPQ, 7‐day SQ‐FFQ and 24HR methods in Mandaluyong City, Philippines^a^.

	Children (12−18 months)				Mothers (18−49 years)			
Method	*n* b	Mean	Median	IQR	*n* b	Mean	Median	IQR
Wheat flour								
FAPQ^c^	114	2.2	0.0	0.0, 3.2	113	5.1	0.0	0.0, 7.6
SQ‐FFQ^d^	113	15.7	11.9	5.5, 21.4	115	51.5	40.4	16.7, 80.5
24HR^e^	113	14.1	12.5	8.3, 18.1	115	42.3	37.6	25.3, 54.2
Oil								
FAPQ^c^	109	4.6	4.0	2.9, 6.2	111	12.5	10.7	7.5, 16.5
24HR^e^	109	1.8	1.6	1.2, 2.2	111	6.1	5.4	3.7, 7.8

Abbreviations: FAPQ, food acquisition and purchase questionnaire; SQ‐FFQ, semiquantitative food frequency questionnaire; 24HR, 24‐h recall.

^a^ Fortifiable was defined as industrially processed and not made at home.

^b^ *n* excludes outliers from FAPQ and SQ‐FFQ methods (values >3 SDs from the mean) and 24HR observations without a corresponding FAPQ or SQ‐FFQ observation.

^c^ Household‐level assessment based on reported amount of fortifiable food the household obtained on the last occasion and duration that this amount usually lasts in the household and application of the adult male equivalent method (Weisell & Dop, 2012).

^d^ Individual‐level assessment based on reported portion size and frequency of consumption of a list of 25 common food items containing fortifiable wheat flour in the past 7 days.

^e^ Individual‐level assessment based on the reported amount of fortifiable food consumed in the past 24 h; mean 5.0 days of data per child (including one 12‐h observed weighed food record plus 12‐h recall in 57 children) and 2.0 days of data per mother; values were adjusted for within‐person variation to estimate usual intakes by applying the amount‐only National Cancer Institute method for estimating usual intake distributions using the Simulating Intake of Micronutrients for Policy Learning and Engagement (SIMPLE) macro (Luo et al., 2021).

### 3.2 Source of fortifiable wheat flour

**Figure 3:**
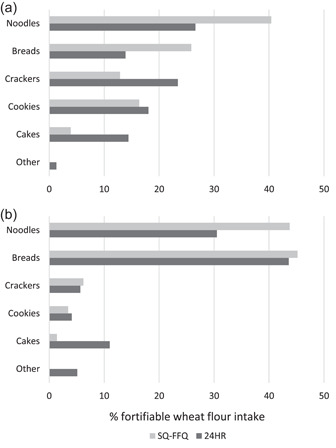
Food sources of fortifiable wheat flour among (a) children (12−18 months) (*n* = 113) and (b) their mothers (18−49 years) (*n* = 115) in Mandaluyong City, Philippines, estimated by 7‐day SQ‐FFQ (individual‐level assessment based on reported portion size and frequency of consumption of a list of 25 common food items containing fortifiable wheat flour in the past 7 days) and 24HR methods (individual‐level assessment based on reported amount of fortifiable food consumed in the past 24 h; mean 3.9 days of data per child and 2.0 days of data per mother). Values indicate percentage of total fortifiable wheat flour consumed (expressed as g/day) derived from each food group based on unadjusted means. ‘Other' includes breaded squid/pork/chicken/sardines and spring roll wrappers (*lumpiang*). SQ‐FFQ, semiquantitative food frequency questionnaire; 24HR, 24‐h recall.

The sources of fortifiable wheat flour (as a percentage of total fortifiable wheat flour consumed from different food groups) estimated by the SQ‐FFQ were generally similar but varied for some food groups when compared to those from the 24‐h recalls (Figure 3). Specifically, the SQ‐FFQ overestimated the percentage of fortifiable wheat flour from noodles and breads among children and noodles among mothers and underestimated that from crackers and cakes among children and cakes among mothers. Additionally, the SQ‐FFQ missed some specific food items that were captured in the 24‐h recalls as breads (i.e., pizza), cakes (i.e., pancakes/waffles, sponge cake [*mamon]*, steamed cake [*puto*], doughnuts, and pastries) and other foods (i.e., breaded squid/pork/chicken/sardines and spring roll wrappers [*lumpiang*]). The ‘other’ category comprised ≤5% of total fortifiable wheat flour consumed, indicating that the SQ‐FFQ captured the major sources of dietary fortifiable wheat flour in this population.

## 4 DISCUSSION

We compared two simplified dietary assessment methods (i.e., a FAPQ and a 7‐day SQ‐FFQ) against 24‐h recalls as the reference method for estimating fortifiable food consumption among young children (12−18 months) and their mothers (18−49 years) in Mandaluyong City, Philippines. According to the 24‐h recalls, mean usual fortifiable wheat flour intake was 14.1 and 42.3 g/day among children and mothers, respectively, and mean usual fortifiable oil intake was 1.8 and 6.1 g/day, respectively. We found that the FAPQ method systematically underestimated fortifiable wheat flour intakes (by more than six times) and overestimated fortifiable oil intakes (by 2−2.5 times) compared to 24‐h recalls while the SQ‐FFQ mean estimates for fortifiable wheat flour were 11%−22% higher, and median estimates were within 5%−7% compared to those from 24‐h recalls.

### 4.1 Findings and implications for fortifiable wheat flour

For fortifiable wheat flour, the substantial and systematic underestimation of intakes by the FAPQ compared to the 24‐h recalls was largely due to the questionnaire excluding measurement of food products made from fortifiable wheat flour that are purchased and/or consumed outside the household. In this study, the FAPQ only captured the quantity of the food item acquired or purchased in its raw form (e.g., as wheat flour obtained by the household). While this works reasonably well in contexts where most foods are prepared in the home, the approach may have limited utility in contexts where prepared food products containing wheat flour are commonly purchased. This was the case in the current study, which took place in an urban setting in the Philippines, where 64% of households reported not using fortifiable wheat flour at home to prepare foods (and thus were assigned zero for amounts consumed), yet the SQ‐FFQ and 24‐h recall results confirmed that wheat flour is commonly consumed as an ingredient in other foods. The most consumed foods containing fortifiable wheat flour were breads and noodles, which are typically purchased already prepared and are not captured by this specific FAPQ version. Other studies in Cameroon, Uganda and Bangladesh that applied the AME method to HCES data on acquisition and purchase of potentially fortifiable wheat flour included questions on both raw wheat flour and common wheat flour‐containing products (which were combined as wheat flour equivalents), but similarly found that the HCES data underestimated total wheat flour consumed compared to reference methods (i.e., 24‐h recalls or observed‐weighed food records) among children under 5 years and women of reproductive age (Dary & Jariseta, 2012; Engle‐Stone & Brown, 2015; Lividini et al., 2013). This suggests there are likely still inaccuracies in measurement with this method, including capturing foods prepared away from home.

On the other hand, the SQ‐FFQ method was successful in capturing the main sources of fortifiable wheat flour in the diets of both children and mothers as compared to the 24‐h recall reference method. However, the SQ‐FFQ had greater variation in the distributions of fortifiable wheat flour intakes compared to the 24‐h recalls. This is likely owing to some missing food items on the food list (as shown in Figure 3), the limited portion size options in the SQ‐FFQ, and/or challenges recalling portion sizes, which may not have captured the true amounts consumed.

### 4.2 Findings and implications for fortifiable oil

For fortifiable oil, the overestimation of intakes by the FAPQ compared to 24‐h recalls is likely due to inaccuracies in the measurement of household utilisation (e.g., difficulty in capturing amounts directly consumed vs. used for cooking) and/or patterns of intrahousehold food distribution that do not correspond to the assumptions of the AME method. The AME method assumes all fortifiable oil acquired is consumed by the members in the household yet in some contexts oil is discarded or reused when preparing foods, which would lead to overestimations of intake. Additionally, the AME method assumes that food is distributed within a household in accordance with the individual's proportion of total household energy requirements based on age and sex. However, this is not always the case, particularly with young children who potentially consume less from the family meals given they are often breastfed and/or receive other complementary foods (Sununtnasuk & Fiedler, 2017). Similar results were observed in studies that applied the AME method to HCES data on acquisition and purchases of fortifiable oil in Cameroon and Bangladesh where mean fortifiable oil intakes among children and women were overestimated compared with reference methods (though median intakes in Cameroon were lower) (Engle‐Stone & Brown, 2015; Lividini et al., 2013) while in Uganda results varied by region (Dary & Jariseta, 2012).

### 4.3 Implications for fortification programme decision making

When using different methods to assess fortifiable food intake, some variation is expected; however, when considering the utility of these methods for fortification programmes the more pertinent question is whether the variation is substantial enough that it would lead to different programmatic decisions. During the programme design phase, fortifiable food consumption data are needed to inform the selection of foods for fortification. In this study, the results from both the SQ‐FFQ and 24‐h recalls would suggest that fortifiable wheat flour is widely consumed and therefore would likely be an appropriate food for fortification. Conversely, the FAPQ results would incorrectly suggest that wheat flour is not likely an appropriate food for fortification in these population groups given its negligible estimated intakes (i.e., median of 0 g/day among both children and mothers). In some cases, fortifiable food consumption data can also be used to inform fortification levels in selected foods (though it is recommended to use them alongside individual‐level data on total nutrient intakes from all foods, beverages and [if applicable] breast milk to ensure fortification levels are set to fill identified nutrient gaps in the population [Coates et al., 2012]). That said, if fortification levels were set using fortifiable food consumption data alone based on the categories of wheat flour intake defined by the World Health Organization (World Health Organization, 2022), the SQ‐FFQ and 24‐h recall results would suggest similar folic acid fortification level recommendations despite the slight variation in results. Both methods resulted in an estimate of <75 g/day wheat flour consumption, which would lead to a recommendation for 5.0 mg/kg of folic acid to be added. The mean amount of wheat flour consumption among mothers would have to be 46% higher than the current estimate to reach the next category of intake (i.e., 75−149 g/day flour consumption) and thus a different folic acid level recommendation (i.e., 2.6 mg/kg). Furthermore, for oil, the FAPQ and 24‐h recalls would both suggest that it is an appropriate food for fortification given it is widely consumed in a fortifiable form. However, the FAPQ method results could potentially lead to lower recommendations for fortification levels compared to 24‐h recalls given its substantial overestimation of daily intakes though there are no specific cutoffs recommended for oil for comparison.

At the programme implementation phase, fortifiable food consumption data can be used to understand fortification programme performance and potential for impact by multiplying intakes by a fortification level (based on actual samples of fortified foods) to estimate additional micronutrient intakes and (if additional data are available on total nutrient intakes from other dietary sources) the extent to which they fill identified gaps in the population. While the range of fortifiable wheat flour intakes varied somewhat between the SQ‐FFQ and 24‐h recalls, the mean and median  estimates did not differ greatly suggesting random error may contribute to observed differences. As a result, the two methods would yield similar mean and median estimates of additional micronutrient intakes and thus similar conclusions regarding average programme performance and potential for impact regardless of the fortification level applied. Conversely, the large and systematic variability between the FAPQ and 24‐h recall intake estimates for both fortifiable wheat flour and oil suggests that true consumption is not likely possible to determine through this method in its current form as additional micronutrient intakes could be greatly over or underestimated. This could result in overly high or low estimates of micronutrient intakes from fortified foods and thus differing expectations about the potential impact of the fortification programme. That said, FAPQ data are likely still better than national food supply data (e.g., food balance sheets), which are often used to estimate potential micronutrient intakes in the absence of individual‐level data, as those do not account for household food acquisition or purchases or permit assessment of subnational variation in use of fortified foods.

### 4.4 Limitations

There were some limitations to this study. The 24‐h recall method was used as the reference method against which the results of the simplified methods were compared; however, it is not a gold standard and has its own sources of error (e.g., recall bias and underreporting) (Hébert et al., 2014). Multiple‐day weighed food records administered over a sufficiently long time frame would be the most accurate dietary assessment method to use as a reference; however, they are even more resource‐intensive than 24‐h recalls and are thus rarely conducted, especially in LMICs. Moreover, the study was conducted in an urban setting in the Philippines; therefore, the results may not be applicable to other settings, particularly rural settings where households prepare most of their foods at home and thus the FAPQ may perform better. Finally, we did not assess total micronutrient intake estimates (from both fortifiable foods and other dietary sources) using the simplified methods and compare them against a reference method in this study. Such information would ideally be used to inform fortification programme decisions, such as setting fortification levels and assessing potential for impact; however, in practice, this information is not often available and data on fortifiable food consumption alone are used.

## 5 CONCLUSIONS

Simplified dietary assessment methods, such as FAPQs and SQ‐FFQs, are alternatives to more detailed methods for generating data on fortifiable food consumption with reduced effort and cost. While this study and others have shown there are still some important differences in the results, the resulting fortification programme decisions may still be similar for some methods and foods (Dary & Jariseta, 2012; Engle‐Stone & Brown, 2015; Lividini et al., 2013). Potential adaptations to the FAPQ, such as additional questions on acquisition and purchase of food products containing fortifiable wheat flour that are prepared away from home and usage patterns for fortifiable oil, merit further research to mitigate its current limitations. Additionally, further research is needed to better understand the sources of error in the application of the AME method to FAPQ data and whether they are unique to some population subgroups (such as young children and women or urban and rural populations) or related to the method itself and its assumptions around utilisation of foods (particularly for oil), and whether questions on complementary food utilisation and/or household distribution may be able to mitigate some of the sources of error. Strengthening simplified dietary assessment methods has the potential to increase the generation of fortifiable food consumption data and enable more evidence‐based decision making in food fortification programmes.

## AUTHOR CONTRIBUTIONS

Valerie M. Friesen, Mduduzi N.N. Mbuya, Lynnette M. Neufeld, Frank T. Wieringa, and Reina Engle‐Stone designed the research; Ame Stormer, Georg Lietz, Marjorie J. Haskell, and Reina Engle‐Stone oversaw implementation of the field study; Jody C. Miller, Ryan B. Bitantes, Maria Fatima Dolly Reario, Mario V. Capanzana, and Carl Vincent D. Cabanilla conducted the research; Valerie M. Friesen, Jody C. Miller, Charles D. Arnold analysed the data; Valerie M. Friesen wrote the paper; Valerie M. Friesen, Charles D. Arnold, Mduduzi N.N. Mbuya, Lynnette M. Neufeld, Frank T. Wieringa, Georg Lietz, Marjorie J. Haskell, Reina Engle‐Stone had primary responsibility for data interpretation; Valerie M. Friesen and Reina Engle‐Stone had primary responsibility for final content; all authors: read and approved the final manuscript.

## CONFLICT OF INTEREST STATEMENT

The authors declare no conflict of interest.

## Data Availability

Data described in the manuscript are available at https://doi.org/10.25405/data.ncl.9771902.v1.
